# *Pseudomonas poae*–Associated Fatal Septic Transfusion Reaction, Peoria, Illinois, USA, 2017

**DOI:** 10.3201/eid2508.181936

**Published:** 2019-08

**Authors:** Therese S. Woodring, John J. Farrell

**Affiliations:** University of Illinois College of Medicine, Peoria, Illinois, USA (T.S. Woodring, J.J. Farrell);; OSF System Laboratory, Peoria (J.J. Farrell)

**Keywords:** transfusion reaction, sepsis, pseudomonas, red blood cell transfusion, psychrophilic bacterium, gram-negative bacteria, bacteria, whole-genome sequencing, cold-tolerant bacteria, *Pseudomonas poae*, fatality, Illinois, United States, transfusion-transmitted infection, septic transfusion reaction

## Abstract

In the United States, fatal transfusion-transmitted infections from red blood cell units are rare. Although this pattern mostly reflects how inhospitable refrigerated red blood cell units are to contaminant growth, fatalities caused by microorganisms that can grow at storage temperature (4°C), but not in standard clinical blood cultures at 37°C, are probably underestimated. We analyzed a fatal red blood cell transfusion in Peoria, Illinois, USA, that occurred in 2017. Samples from the patient’s whole blood and the red blood cell unit remained culture-negative during the investigation, despite direct visualization of gram-negative bacilli within the unit immediately after transfusion. We identified the bacteria as *Pseudomonas poae*, a nonpathogenic pseudomonad carrying multiple cold-shock domain protein genes, and confirmed its cold tolerance and inability to grow at 37°C. Our work indicates transfusion reaction workups need to include testing for psychrophilic organisms, which could explain the cause of other apparently culture-negative transfusion reactions.

 Transfusion-transmitted infections (TTIs; i.e., the transmission of bacteria, viruses, parasites, or prions through blood product transfusions) are reportable events (*1*). Although >5 million patients in the United States receive red blood cell transfusions each year, fatal TTIs from contaminated red blood cell units number in the single digits annually and arise primarily from *Babesia* infection in the donor (Table 1). Bacterial colonization of the red blood cell unit is a much rarer event, and viruses and prions, which are the target of most donor history questionnaires and blood product screening tests, have not contributed to reported fatalities in the past decade. The rarity of bacterial contamination reflects vigilant collection practices for all blood products, including skin disinfection and diversion of the first few milliliters of blood from healthy donors, as well as red blood cell refrigeration, which further decreases contamination risk compared with nonrefrigerated blood products, like platelets.

**Table 1:** Fatalities caused by red blood cell transfusions reported to the US Food and Drug Administration, 2005–2016*

Year	No. fatalities	Organisms (no.)
2005	1	*Serratia marcescens*
2006	4	*Babesia microti* (n = 2), *Escherichia coli* (n = 1), *Yersinia enterocolitica* (n = 1)
2007	3	*B. microti*
2008	5	*B. microti*
2009	0	
2010	1	*B. microti*
2011	1	*B. microti*
2012	1	*B. microti*
2013	2	*B. microti* (n = 1), *Pseudomonas fluorescens* (n = 1)
2014	0	
2015	1	*Enterococcus faecium*
2016	3	*B. microti* (n = 2), *P. fluorescens* (n = 1)
All	22	*B. microti* (n = 16), *P. fluorescens* (n = 2), *E. coli* (n = 1), *Y. enterocolitica* (n = 1), *S. marcescens* (n = 1), *E. faecium* (n = 1)

*See (*2*,*3*).

Still, the rate of fatalities resulting from red blood cell contamination, particularly by bacteria, is surprisingly low, given that, unlike platelets, bacterial contamination of red blood cell units is not screened for after collection, apart from a serologic test for syphilis and visual inspection for gross contamination immediately before transfusion (*4*). Moreover, pathogen inactivation technologies used after collection for plasma and platelets are not yet available for red blood cells (*5*). This low rate of bacterial contamination might partly reflect how inhospitable the red blood cell unit becomes to contaminating organisms over its 42-day shelf life. Held at only a few degrees above freezing (4°C), the unit becomes progressively depleted of high-energy substrates, and waste products and reactive oxygen species accumulate at a pH well below the physiologic pH range for blood (*6*,*7*). Bacteria that can grow to life-threatening numbers in this environment must be capable of surviving these conditions, and the risk for contamination with these organisms within collection facilities is rare enough to pose minimal threat to the blood supply.

Organisms adapted for survival in packed red blood cell units during storage, however, could be missed during evaluations of suspected transfusion reactions in the clinical laboratory by virtue of their specialized growth limitations. For instance, bacteria best suited to survive in a refrigerated red blood cell unit might not grow at 37°C, the standard temperature for incubation of suspected TTI workups. Yet, these bacteria do not need to grow at this temperature to trigger a fatal septic reaction upon infusion if their inoculum size is large and endotoxin concentration high. The Centers for Disease Control and Prevention National Healthcare Safety Network guidelines for definite TTIs require evidence of the infectious agent in the transfused unit or recipient (Table 2) (*8*). When standard laboratory testing is used alone, these cases involving cold-tolerant bacteria could be excluded from fatality statistics for TTIs. We present a case report of a death occurring after transfusion with a contaminated red blood cell unit; the investigation required advanced techniques, such as whole-genome sequencing (WGS), to determine the colonizing agent.

**Table 2:** Centers for Disease Control and Prevention National Healthcare Safety Network criteria for establishing definite transfusion-transmitted infections*

Criteria
>1 of the following:
Evidence of the pathogen in 1) the transfused component, 2) the donor at the time of donation, 3) an additional component from the same donation, or 4) an additional recipient of a component of the same donation
AND
No other potential exposures to the pathogen be identified for the recipient
AND
Either evidence that the recipient was not infected with the pathogen before transfusion or evidence that the identified pathogens are related by molecular or extended phenotypic comparison testing

*See (*8*).

## Clinical Case

**Figure 1:**
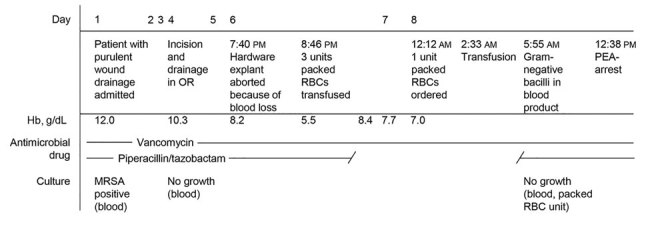
Timeline of patient’s hospitalization for periprosthetic joint infection, followed by fatal septic transfusion reaction, Peoria, Illinois, USA, 2017. Hb, hemoglobin; MRSA, methicillin-resistant *Staphylococcus aureus*; PEA, pulseless electrical activity; RBC, red blood cell.

In 2017, a 56-year-old woman with a history of diabetes mellitus, hypertension, and right femur fracture requiring open reduction and internal fixation with total knee arthroplasty (TKA) was admitted to a hospital in Peoria, Illinois, USA, with leg pain and inability to walk. Her TKA had been complicated 5 months earlier by periprosthetic femur fracture and infection with *Corynebacterium striatum* and *Pseudomonas aeruginosa*, which was treated with intravenous vancomycin and cefepime. At admission, she was noted to have purulent drainage from an open right thigh wound above a long plate in her femur (Figure 1), and blood and wound cultures were positive for methicillin-resistant *Staphylococcus aureus*. She was immediately started on intravenous vancomycin, and operative incision and drainage were performed on day 4 of hospitalization without complications. By the day the TKA prosthetic was scheduled for removal (day 6), her blood cultures were negative for bacterial growth.

On day 6, medical staff attempted explant of the TKA hardware; however, the procedure needed to be aborted because of excessive blood loss during debridement. Her preoperative hemoglobin concentration dropped from 10.3 g/dL to 5.5 g/dL within 1 hour of the procedure. She received 3 units of typed and crossed packed red blood cells without complication. Five hours after this transfusion, her hemoglobin rose to 8.4 g/dL, but a repeat hemoglobin assessment 21 hours later indicated the concentration dropped (7.0 g/dL), so another red blood cell unit was ordered for her.

Within 5 minutes of starting the final transfusion, the patient became tachypneic; tachycardia developed, and she began to report shortness of breath. According to hospital protocol, the transfusion was stopped immediately. Clerical error and a hemolytic transfusion reaction were excluded by repeat donor ABO typing, a direct antiglobulin test, and visual inspection of plasma and urine for hemolysis. The red blood cell unit had no signs of hemolysis or breached bag integrity. Despite empiric treatment for an allergic transfusion reaction, the patient continued to exhibit signs of a systemic inflammatory response (heart rate 120–140 beats/min, respiratory rate 35–40 breaths/min) and required increasing oxygen supplementation from a nonrebreather mask and bilevel positive pressure ventilation. Three hours after the transfusion, the laboratory reported gram-negative bacteria throughout smears taken from the red blood cell unit as part of laboratory protocol for suspected transfusion reactions. The patient was started on intravenous piperacillin/tazobactam but continued to deteriorate, undergoing 4 episodes of cardiopulmonary resuscitation before she was declared dead of cardiac arrest 10 hours after the transfusion. Standard 37°C clinical blood cultures from both the patient and the red blood cell unit were negative for bacterial growth after 5 days. The transfusion reaction was reported to the Food and Drug Administration 6 days after the patient’s death.

## Methods

For blood culture testing, we inoculated a BACTEC Peds Plus (BD, https://www.bd.com) blood culture bottle with 1.0 mL of blood from the red blood cell unit and left the sample at ambient temperature (25°C) for 24 h. Using this culture, we streaked organisms onto a blood agar purity plate and incubated for 24 h at 25°C. We spotted 1 purified colony for matrix-assisted laser desorption/ionization time-of-flight (MALDI-TOF) mass spectrometry (VITEK MS; bioMérieux, https://www.biomerieux-diagnostics.com) and suspended another colony from the same plate in sterile saline of 0.99 McFarland turbidity standard in preparation for WGS. We extracted DNA from the suspended colony using the QIAGEN REPLI-g UltraFast Mini Kit (https://www.qiagen.com) according to manufacturer instructions and sequenced with a FLO-MIN 106 SpotON Flow Cell on a Nanopore MinION Mk1B (Oxford Nanopore Technologies, https://nanoporetech.com) using a rapid whole-genome amplification protocol (SQK-RAD004).

We analyzed reads passing the default quality score cutoff of 7 for quality in NanoPlot (*9*) and assembled de novo using Canu version 1.7.1, an assembler designed for long-read output (*10*). We evaluated the assessment of the de novo assembly quality using Bandage (*11*). We retained contigs with >5× coverage for further error correction using Nanopolish 0.8.5 with default settings (*12*) and annotated the final assembly with Prokka 1.13.3 (*13*). We identified the species by analyzing the 16S rRNA sequence using the Ribosomal Database Project Seqmatch tool and blastn (https://blast.ncbi.nlm.nih.gov/Blast.cgi) within the National Center for Biotechnology Information database. We performed multilocus sequence analysis with 4 conserved gene regions previously used for *Pseudomonas* taxonomy (16S-*gyrB*-*rpoB*-*rpoD*) on the isolate assembly and 20 fluorescens subgroup genomes available in GenBank (Table 3) (*14*). For multilocus sequence analysis, we aligned each gene region with MUSCLE before concatenation in Geneious 11.1.5 (*15*).

**Table 3:** *Pseudomonas* spp. included in multilocus sequence analysis to identify bacterial contaminant in red blood cell unit, Peoria, Illinois, USA, 2017

Species	Strain	GenBank accession no.
*P. poae*	RE*1–1-14	GCA_000336465.1
*P. azotoformans*	S4	GCA_001579805.1
*P. extremorientalis*	BS277	GCA_900104365.1
*P. simiae*	WBS417	GCA_000698265.1
*P. palleroniana*	MAB3	GCA_002953635.1
*P. tolaasii*	2192T	GCA_002072675.1
*P. costantinii*	LMG 22119	GCA_001870435.1
*P. antarctica*	PAMC 27949	GCA_001647715.1
*P. fluorescens*	F113	GCA_000237065.1
*P. salomonii*	ICMP 14252	GCA_900107155.1
*P. trivialis*	IHBB745	GCA_001186335.1
*P. rhodesiae*	BS2777	GCA_900105575.1
*P. marginalis*	ICMP 9505	GCA_001467265.1
*P. panacis*	BS2778	GCA_900104875.1
*P. grimontii*	BS2976	GCA_900101085.1
*P. veronii*	R02	GCA_002028325.1
*P. cedrina*	BS2981	GCA_900104915.1
*P. orientalis*	F9	GCA_002934065.1
*P. libanensis*	BS2975	GCA_900101035.1
*P. synxantha*	LBUM223	GCA_000968415.2
*P. aeruginosa*	PAO1	GCA_000006765.1

After WGS analysis, we analyzed temperature-dependent viability by incubating isolates from the original blood agar purity plate in BD BACTEC Peds Plus blood culture bottles at 4°C, 25°C, and 37°C for 5 days. We plated cultures diluted 1:100–1:10,000,000 on blood agar and counted the colonies that grew after 24 h at 25°C.

## Laboratory Investigation

Initial isolate identification by MALDI-TOF mass spectrometry was split between *Pseudomonas fluorescens* (50% confidence) and *P*. *veronii* (50% confidence). WGS produced a total of 168,870 reads spanning 885,192,362 bp that passed the quality threshold. The median read length was 3,200 (maximum 100,184) bp. The assembly contained 2 contigs: a 7,340,165-bp contig with 23.4× coverage corresponding to the predicted chromosomal length of *Pseudomonas* spp. (GenBank accession no. CP034537) and a 150,410-bp contig with 41.6× coverage suggestive of a plasmid (GenBank accession no. CP034538).

**Figure 2:**
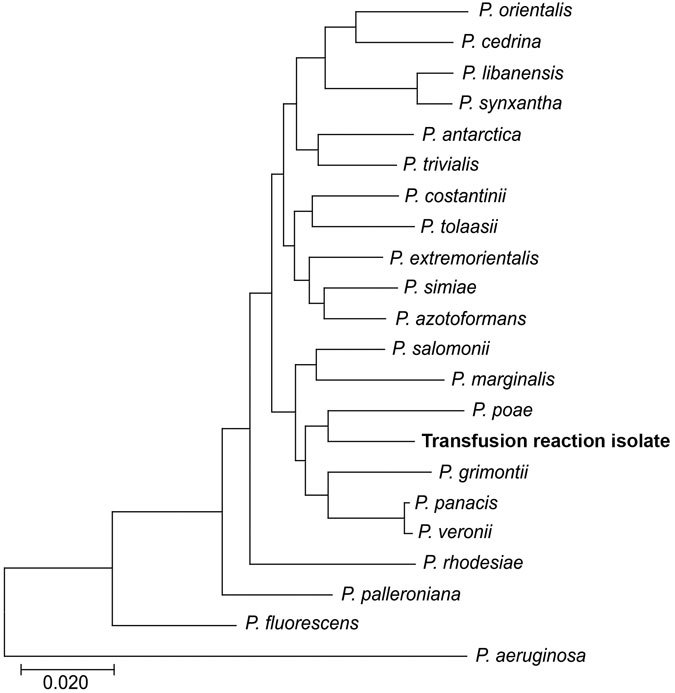
Neighbor-joining tree calculated by using Jukes-Cantor model for concatenated 16S-*gyrB*-*rpoB*-*rpoD* gene sequences of the *Pseudomonas* isolate from patient in Peoria, Illinois, USA, 2017 (bold), and 20 fluorescens subgroup pseudomonads. We used *P. aeruginosa* as the outlier. Scale bar indicates nucleotide substitutions per site.

Among the 8,602 genes annotated on the chromosome were 6 full-length 16S rRNA genes, consistent with the higher 16S gene copy numbers seen in the *P*. *fluorescens* and *P*. *putida* clusters (n = 5–7) compared with the copy number of *P. aeruginosa* clusters (n = 4) (*16*). The sequence of this gene matched with >99% similarity to that of *P. poae*, a fluorescens group pseudomonad, by both the Ribosomal Database Project Seqmatch tool and blastn (Table 4). In multilocus sequence analysis (16S-*gryrB*-*rpoB*-*rpoD*), the isolate also clustered with *P*. *poae*, further supporting this identification (Figure 2).

**Table 4:** Identification of bacterium in red blood cell unit, Peoria, Illinois, USA, 2017, on the basis of 16S rRNA gene sequencing results, by database

Database	Identification	Metric
Ribosomal Database Project	*Pseudomonas* sp. VS05_16	1.000 similarity score
	*P. poae* BCHCBZ253	0.996 similarity score
GenBank	*P. poae* strain BA2776	100% coverage, 99% identity (1,530/1,532 nt)
	*P. poae* RE*1–1-14	100% coverage, 99% identity (1,530/1,532 nt)

**Figure 3:**
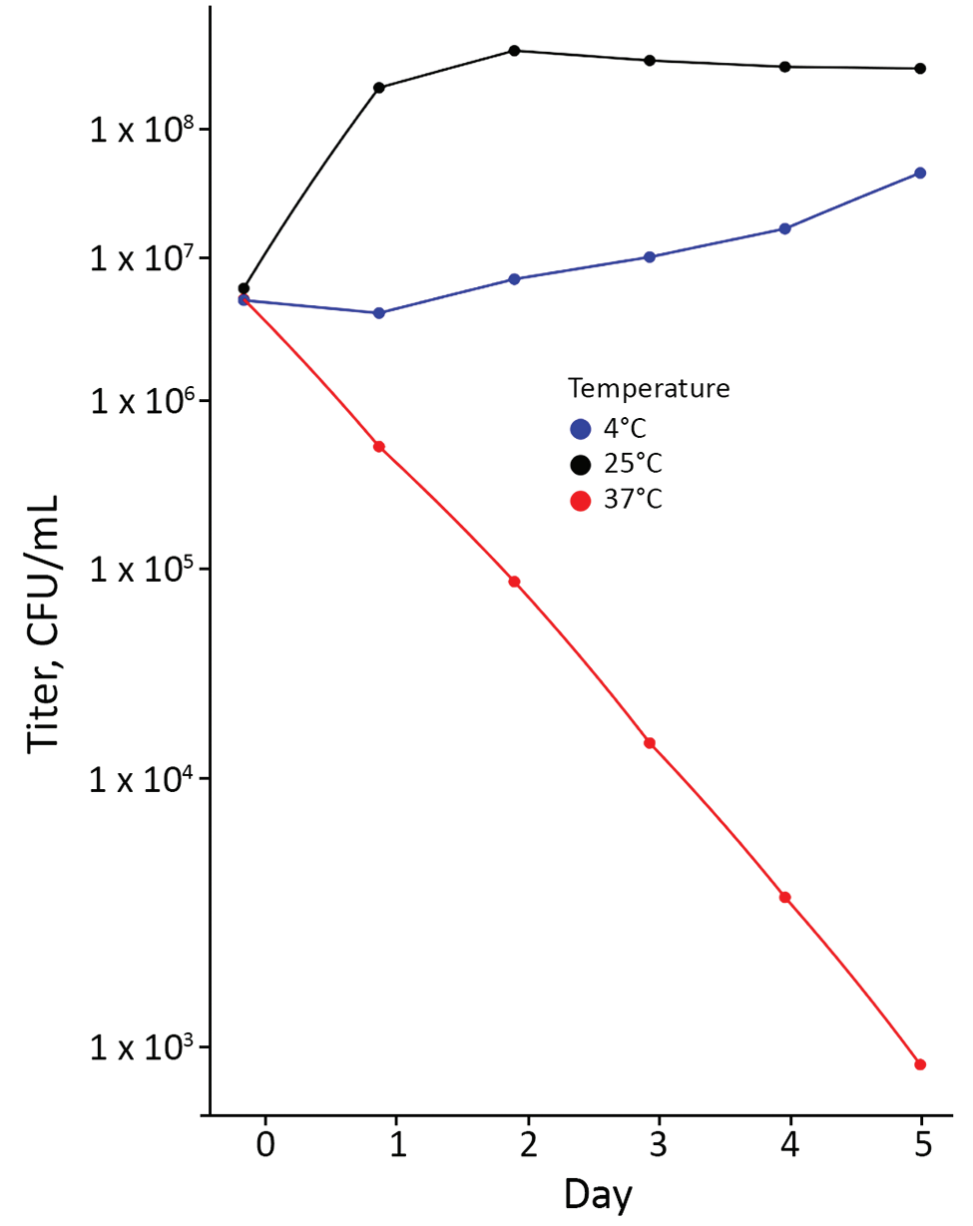
Titers of viable bacteria in cultures of *Pseudomonas poae* from patient in Peoria, Illinois, USA, 2017. Cultures were grown in BD BACTEC Peds Plus (https://www.bd.com) blood culture media incubated at 4°C, 25°C, and 37°C for 5 days.

**Figure 4:**
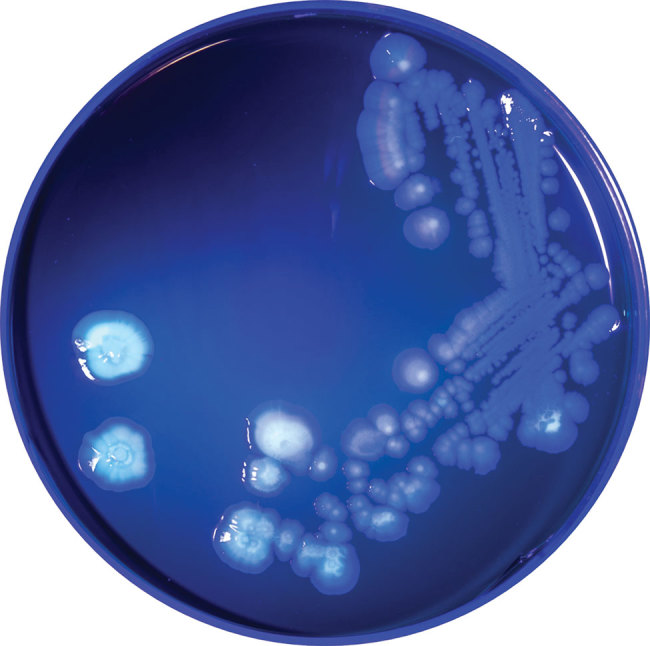
*Pseudomonas poae* colonies isolated from contaminated packed red blood cell unit, Peoria, Illinois, USA, 2017, fluorescing under ultraviolet light (λ = 395 nm).

The annotated genome contained multiple copies of *capB* and *cspA*, genes for cold-shock domain proteins that enable efficient translation and long-term cold adaptation in Antarctic pseudomonads (Table 5) (*17*). Correlating this genomic signature with phenotypic data, we conducted a temperature-dependent viability experiment that confirmed growth at 4°C and 25°C (Figure 3) and no growth at 37°C; >99.9% of bacteria died within 5 days of incubation at 37°C. In addition, the annotated genome included evidence of versatile iron-acquisition capacities, including 2 siderophore systems and extracellular heme scavenging (Table 5) (*18*,*19*). Fluorescent siderophores are responsible for the fluorescence that gives the fluorescens group its name. Our isolate did demonstrate fluorescence under ultraviolet light, consistent with siderophore production (Figure 4).

**Table 5:** Genes in *Pseudomonas* *poae* from contaminated red blood cell unit, Peoria, Illinois, USA, 2017, that potentially facilitated its survival in the red blood cell unit stored at 4°C*

Gene	Product	Function
*cspA*	Major cold shock protein CspA	Maintains efficient translation at low temperatures
*capB*	Cold shock protein CapB	Permits long-term cold growth
NRPS clusters	Pyoverdine (nontemplated siderophore)	Synthesis of siderophore pyoverdine and cognate receptor used for iron acquisition
*fpvA*	Ferripyoverdine receptor	
*entD*	Enterobactin synthase	Synthesis of secondary siderophore enterobactin and cognate receptor used for iron acquisition
*pfeA*	Enterobactin receptor	
*hasA*	Hemophore HasA	Extracellular heme scavenging protein

*NRPS, nonribosomal peptide synthetase.

## Discussion

WGS analysis identified the organism within the red blood cell unit from this case as *P*. *poae*, a cold-adapted fluorescens pseudomonad first discovered in the grass phyllosphere in 2003 and since found around the world, including the cold deserts of the Himalayas (*20*,*21*). Previous studies of this organism have focused on its potential to promote plant growth through phosphate mobilization, its production of plant-protective antifungal metabolites (*22*,*23*), and its ability to remediate contaminated ecosystems through hydrocarbon degradation (*24*). A Medline search yielded no reports of *P*. *poae* as a human pathogen. Considering how poorly this isolate tolerated body temperature (37°C), the lack of clinical cases might reflect a genuinely low virulence in humans.

The pathogenicity of this organism seems specific to the ecology of red blood cell storage and transfusion. On both a genomic and phenotypic level, we found evidence of cold tolerance that particularly suited *P*. *poae* to not just surviving but thriving in long-term refrigeration in a red blood cell unit. Because the organism grew at 25°C, any length of improper red blood cell storage at room temperature could have provided additional opportunity for growth. The versatile iron acquisition capacities suggested by genome annotation indicate the potential for *P*. *poae* to exploit iron within the red blood cell unit environment (*25*). Although we cannot specify where the opportunity for colonization arose, *Pseudomonas* spp. have been traced to environmental sources, including contaminated water baths and cooling cloths in septic episodes involving blood products (*26*,*27*). *Pseudomonas* spp. are also common reagent contaminants detected in sequencing-based studies (*28*), suggesting that donor skin and the environment are not the only possible sources of contamination.

Once *P*. *poae* was introduced into the patient, its survival was likely not required for virulence. Given a sufficiently large inoculum, the endotoxin and other antigens from both live and dead organisms that accumulated during the unit’s storage could have provided enough of an immunogenic stimulus within the bloodstream to trigger a massive dysregulated immune response, irrespective of the ability of the organism to establish a sustained infection at 37°C (*29*). Indeed, endotoxin is sufficient to activate multiple innate immune pathways that contribute to the hemodynamic, metabolic, and coagulation defects driving death due to sepsis (*30*,*31*). In the case we describe, how the history of methicillin-resistant *S*. *aureus* sepsis <1 week before the infection affected the patient’s risk for death is unclear; systemic weakening or modification of the subsequent immune response could have been contributing factors that affected the patient’s outcome. Studies of sepsis in animals with previous endotoxin exposure suggest diverging effects of this priming on the basis of the dose (*32*,*33*); in vitro, cell wall components of gram-positive bacteria (e.g., lipoteichoic acid) appear to potentiate cytokine responses at low doses of endotoxin and suppress them at high doses (*34*).

National Healthcare Safety Network criteria for a definite TTI are predicated on pathogen identification, which requires not only a Gram stain, as done in this case, but also a positive blood culture and subsequent isolation on solid media (*8*). Although standard laboratory testing alone did not meet National Healthcare Safety Network criteria for a definite TTI in this case, we believe the presence of the *P*. *poae* isolate in the transfused blood product was the cause of death for this patient. The patient’s clinical deterioration from hemodynamic stability to death by cardiovascular collapse progressed during the 10 hours immediately after transfusion, and WGS provided postmortem evidence of *P*. *poae* in the red blood cell unit. Although *P*. *poae* is ubiquitous in soil, this patient had no other plausible systemic exposures to this otherwise nonpathogenic organism. In addition, the patient becoming infected with *P*. *poae* before transfusion was overwhelmingly unlikely; she had received a 6-day course of empiric piperacillin/tazobactam covering gram-negative organisms for her periprosthetic infection within 48 hours of the transfusion, and her negative blood cultures before transfusion make any preexisting bloodstream infection with any *Pseudomonas* spp. highly improbable. Given that we believe the organism caused the patient’s death without establishing infection, the more appropriate term in this case would be septic transfusion reaction rather than TTI.

Because of the rapid death of this patient, a patient-derived blood sample was not accessible for next-generation sequencing and endotoxin testing, limiting our study. Because *P*. *poae* is a ubiquitous plant- and soil-associated organism, we also cannot definitively exclude that the isolate we sequenced was not a contaminant from postmortem handling of the red blood cell unit. However, the absence of these gram-negative organisms (which were visible in blood smears acquired from the blood bag within 5 hours after transfusion) in the standard 37°C blood culture is consistent with the temperature intolerance observed for the gram-negative isolate later recovered from the red blood cell unit. Moreover, the growth of this isolate at 4°C coupled with the observation that most fatal bacterial TTIs are caused by cold-tolerant gram-negative organisms (e.g., *Serratia* spp., *Yersinia enterocolitica*) suggests that the isolate we recovered originated from the refrigerated red blood cell unit (Table 1) (*35*).

Our case expands the literature on microbially mediated deaths from red blood cell transfusions and represents an extraordinary human fatality from *P*. *poae*. This episode draws attention to the limitations of standard blood culture procedures to fulfill National Healthcare Safety Network TTI criteria, which, for all practical purposes, require that organisms be incubated under conditions that much more resemble the human body than the cold storage environment selecting for the contaminating organisms. *P*. *poae* is a prime example of an organism that could, by virtue of the very temperature-dependent growth that enables its survival in refrigerated red blood cell units, elude detection in a transfusion reaction investigation. Until guidelines expand to include cultures for bacteria that grow at storage temperature, the number of apparently culture-negative adverse transfusion reactions that are caused by similar organisms will remain unknown. Finally, our case demonstrates the potential for next-generation sequencing to detect, identify, and characterize organisms directly from contaminated blood products.
